# Exercise Training Improves Memory Performance in Older Adults: A Narrative Review of Evidence and Possible Mechanisms

**DOI:** 10.3389/fnhum.2021.771553

**Published:** 2022-01-27

**Authors:** Parvin Babaei, Helya Bolouki Azari

**Affiliations:** ^1^Neuroscience Research Center, School of Medicine, Guilan University of Medical Sciences, Rasht, Iran; ^2^Cellular and Molecular Research Center, School of Medicine, Guilan University of Medical Sciences, Rasht, Iran; ^3^Department of Physiology, School of Medicine, Guilan University of Medical Sciences, Rasht, Iran; ^4^Department of Physiology, Tehran University of Medical Sciences, Tehran, Iran

**Keywords:** exercise training, neurogenesis, neurotrophic factors, myokines, memory, adipokines

## Abstract

As human life expectancy increases, cognitive decline and memory impairment threaten independence and quality of life. Therefore, finding prevention and treatment strategies for memory impairment is an important health concern. Moreover, a better understanding of the mechanisms involved underlying memory preservation will enable the development of appropriate pharmaceuticals drugs for those who are activity limited. Exercise training as a non-pharmacological tool, has been known to increase the mean lifespan by maintaining general body health and improving the cardiovascular and nervous systems function. Among different exercise training protocols, aerobic exercise has been reported to prevent the progression of memory decline, provided adequate exertion level, duration, and frequency. Mechanisms underlying exercise training effects on memory performance have not been understood yet. Convergent evidence suggest several direct and indirect mechanisms at molecular and supramolecular levels. The supramolecular level includes improvement in blood circulation, synaptic plasticity and neurogenesis which are under controls of complex molecular signaling of neurotransmitters, neurotrophic factors, exerkines, and epigenetics factors. Among these various factors, irisin/BDNF signaling seems to be one of the important mediators of crosstalk between contracted skeletal muscles and the brain during exercise training. This review provides an affordable and effective method to improve cognitive function in old ages, particularly those who are most vulnerable to neurodegenerative disorders.

## Graphical Abstract

Exercise, neurotransmitters, growth factors, myokines, and potential effects on the brain.

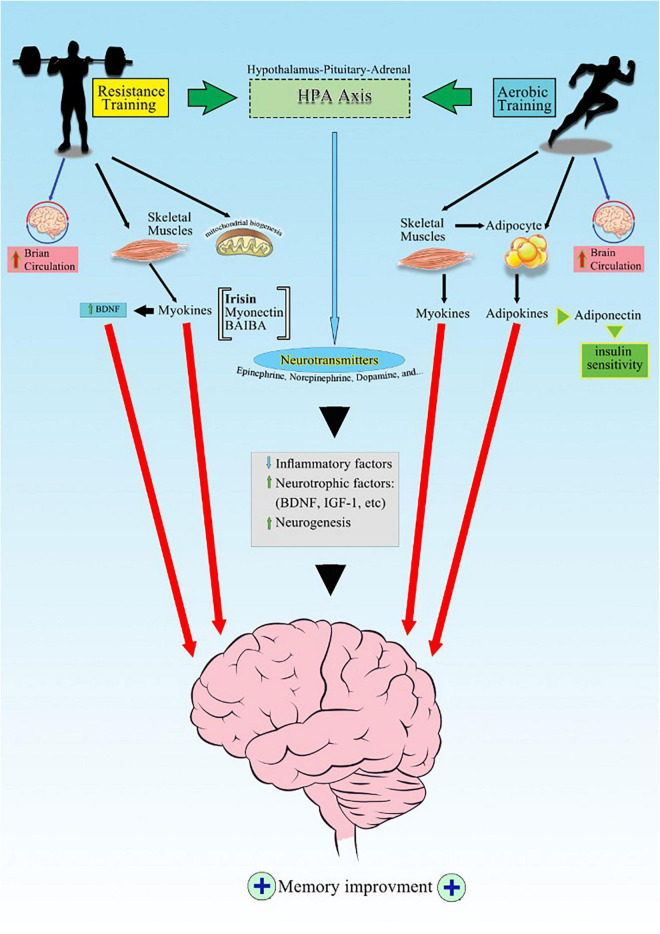

## Highlights

- Exercise training enhances memory performance via neuroplastic alterations.
- Long-lasting moderate aerobic exercise is a more efficient neuroprotective modality.
- Exercise-induced memory improvement might be mediated via neurotrophic factors, and exerkines.
- Irisin/BDNF signaling is an important link between skeletal muscles and the brain.

## Introduction

### Epidemiological Overview

Cognitive function has been known to be negatively associated with aging (Plassman et al., 2007), genetic predisposition, cardiovascular disease, and type 2 diabetes (Grøntved and Hu, 2011). Currently, 35.6 million people worldwide live with dementia which is predicted to double to 75.6 million by 2030, thus, soon cognitive deficit will be a major public health priority (World Health Organization, 2020). The reasons responsible for the cognitive deficit are not yet well established. However, some assumptions have arisen, and among them, the reduction in the speed of information processing, sensorial deficit, decline in learning, and memory capability due to aging are more prominent (Ball and Birge, 2002; Kramer and Willis, 2002).

On the other hand, exercise training as a healthy lifestyle factor reduces the likelihood of developing dementia or slow down the progress of cognitive decline both in normal aging and dementia (Larson et al., 2006; Rolland et al., 2007; Chang et al., 2010, 2012; Buchman et al., 2012; World Health Organization, 2019; Lewis et al., 2020). It has been reported that engagement in exercise training by 5% over 5 years, reduces the percentage of patients with dementia by 11% (Grøntved and Hu, 2011).

Considering the remarkable evidence indicating positive association between exercise training and cognitive function, the American College of Sports Medicine (ACSM) and the American Heart Association (AHA) recommended daily exercise for adult individuals (Voss et al., 2010).

## Cognitive Function

Cognitive function refers to higher-level functions of the brain and includes different modalities such as acquiring knowledge, perception, attention, judgment, decision making, processing speed, executive function, cognitive flexibility, task switching, comprehension, response inhibition, and memory performance (Lezak et al., 2004; Diamond, 2013). Cognitive flexibility is an ability associated with adjusting mental activity and content, switching between different task rules and corresponding behavioral responses, maintaining multiple concepts simultaneously, and shifting internal attention between them to make a better adaptation to a new context (Scott, 1962; Cooper-Kahn and Laurie, 2008). Also, the ability to simultaneously consider two aspects of an object, idea, or situation at one point in time refers to cognitive flexibility, which requires aspects of inhibition, attention, working memory, response selection, and goal maintenance (Miyake et al., 2000; Sheet, 2005). Cognitive flexibility is mediated via a complex network including the front parietal cortex, cingulate cortex, mesolimbic, and striatum (ventral and dorsal parts) (Nowrangi et al., 2014; Hall and Fong, 2015). Considering the complexity of cognitive flexibility elements, the related neural networks, and limitation in the existing literature, we decided to review the effects of exercise training only on memory; as a vital component of cognitive flexibility.

The memory is an exciting capability of brain which preserves and stores acquired information and enables performing of adequate behavior based on lifelong experience (Magila and Xavier, 2012). Any deficits in memory retrieval might have deleterious implications on individual routines and health (Colcombe and Kramer, 2003).

Therefore, the arguments developed in this review focused on exercise training’s effects on memory performance and is not intended to cover all aspects of cognitive function.

### Exercise Effects on Memory Performance

Exercise training is defined as planned, structured, and repetitive exercise (Caspersen et al., 1985). There are remarkable pieces of evidence indicating that regular exercise training slows down the progress of cognitive decline (Erickson et al., 2011; World Health Organization, 2019; Lewis et al., 2020), and maintain the brain’s cognitive ability, particularly memory, however, there are inconsistencies in the literature due to the variety in type and timing of the cognitive tests, subjects characteristics (Sibley et al., 2006; Lambourne and Tomporowski, 2010; Chang et al., 2012), and exercise protocols (Loprinzi et al., 2019a).

Some researchers believe that working memory is improved by chronic exercise, but not acute, in elderly individuals (Smith et al., 2010; Rathore and Lom, 2017; Damirchi et al., 2018), elite soccer players (Babaei et al., 2014); and in Alzheimer’s disease patients (Vreugdenhil et al., 2012). Both animal study in aged rats with the Parkinson’s disease (Tsai S.-F. et al., 2018; Tsai et al., 2019), and human study in patients diagnosed with Alzheimer’s disease (Jia et al., 2019) and also in healthy adults (Babaei et al., 2014), recommended engaging in exercise training for 16–24 weeks, at least up to 3 times per week with 30 min per session, in order to achieve better outcomes on working memory. Liu et al. (2020), in patients with dementia confirmed that both strength aerobic and resistance training programs over 4 weeks can bring about significant cognitive benefits. In contrast with chronic protocols, Loprinzi et al. (2019a) reported that acute moderate-intensity exercise prior to memory encoding is capable to enhance short and long-term memories in healthy individuals (Loprinzi et al., 2019a). The relationship between acute exercise and memory is complex to conclude, and may vary based on the temporality, intensity of exercise, and the memory type evaluated.

Taken altogether, regular chronic type of training is more prominent for memory facilitation.

In the next section we discuss about the effect of intensity of training on memory performance.

### The Effect of Exercise Intensity on Memory Performance

Besides the modalities of exercise training discussed above, the intensity of exercise training in relation to memory tests is important as well. Some studies suggest a dose-effect relationship between aerobic activity and executive function (Masley et al., 2009; Pyke et al., 2020), but, some believe in a reverse u shape pattern, meaning that a moderate-intensity exercise would improve memory, whereas high-intensity exercise would impair memory performance (Brisswalter et al., 2002; Kashihara et al., 2009; Ruscheweyh et al., 2011; Frith et al., 2017).

It should be noticed that, the timing of memory tests is very important too. Loprinzi et al. (2019b) reported that acute moderate-intensity exercise prior to memory encoding enhances short and long-term memories in healthy participants (Loprinzi et al., 2019a), however high-intensity acute exercise impairs working and episodic memories (Loprinzi et al., 2019b).

In addition, a decline in cognitive performance of adults, when measured during acute high-intensity physical exercise (Isaacs, 1991; McMorris and Keen, 1994), or immediately after the exercise (Covassin et al., 2007; Bue-Estes et al., 2008; Moore et al., 2012) have been found. Moreover, the declines in working memory, verbal memory, and attention have been shown to be transient following intense activity in adults (Covassin et al., 2007; Bue-Estes et al., 2008; Moore et al., 2012), and children (Samuel et al., 2017).

A recent study reported that a single bout of maximal intensity exercise in children can transiently impair complex tasks such as verbal learning, which resolves after a 1-h rest. On the other hand, more simple cognitive tasks that apply short-term working memory are not negatively affected by such an activity, and may even be facilitated after adequate rest (Samuel et al., 2017). Therefore, adequate recovery from intense exercise might result in enhanced working memory abilities (Bue-Estes et al., 2008), indicating both debilitating (Covassin et al., 2007; Bue-Estes et al., 2008; Moore et al., 2012) and facilitating (Bue-Estes et al., 2008) effects of physical activity.

Overall, when high-intensity acute exercise occurs before or during the memory task, it may be less favorable for working memory, and may not associate with long-term memory function, as opposed to when it occurs shortly after memory encoding (Hötting et al., 2016; Loprinzi, 2018).

Also, exercising shortly after memory encoding is slightly less advantageous for both moderate (Labban and Etnier, 2011; Sng et al., 2018) and high-intensity exercise (Frith et al., 2017) compared to pre encoding.

Interestingly, in contrast with the negative relationship between high-intensity exercise and memory performance which discussed above, Wilke (2020) believes that high-intensity functional training represents an appropriate method to acutely improve working memory, in healthy middle-aged individuals. One of the explanations for discrepancy in findings possibly related to the fatigue which plays a more important role on the cognitive responses to an exercise bout.

Collectively, there is an intensity-specific effect of exercise on memory, and results may differ based on the memory tests, the temporality of memory assessment, the time elapsed from completion of the exercise, and the method of estimating the intensity of exercise training.

Additional works exploring the effects of a variety of exercise types (e.g., continuous, intermittent, strength, aerobic, combined-type) under different intensities and durations upon memory storage and retrieval are needed.

### Possible Mechanisms of Exercise-Induced Memory Improvement

Although mechanisms underlying exercise training effects on memory improvement have not been understood well, there are both direct and indirect mechanisms at molecular and supramolecular levels. The supramolecular level refers to the alteration in the physiological functions or structural changes in organs such as increase in blood circulation or hippocampal volume (Colcombe and Kramer, 2003; Smith et al., 2010; McAuley et al., 2011; Guiney and Machado, 2013). The supramolecular alterations, *per se* are under the control of complex molecular signaling pathways. For instance, increasing gray matter integrity, and hippocampal volume (Colcombe and Kramer, 2003; Smith et al., 2010; McAuley et al., 2011; Guiney and Machado, 2013) are mostly related to neurogenesis (Sibley et al., 2006; Chang et al., 2012; Foster, 2015) and elevation in neurotrophic factors (Hötting et al., 2016).

Below, we discuss in more detail some of the selected molecular mechanisms underlying exercise’s beneficial effects on learning and memory.

#### Brain Circulation

Regular physical activity has been reported to improve brain circulation (Erickson et al., 2012) particularly the hippocampus; an area important for learning and memory (Pereira et al., 2007; Burdette et al., 2010; Mandolesi et al., 2017). How exercise leads in elevated circulation and further memory improvement, has not been clarified yet. It is assumed that skeletal muscles induces the secretion of lactate, during contraction, then, lactate is taken up by the brain regions (Ide and Secher, 2000), and causes excitability of the primary motor cortex (Coco et al., 2010), increases brain vascular endothelial growth factor (Morland et al., 2017) and density of cerebellar cortex vessels (Castellano et al., 2017). Besides providing adequate pumping and oxygenation of the blood, lactate increases brain metabolism by ketone uptake and utilization.

Adequate circulation also provides clearance of the brain waste products such as amyloid-beta; an important abnormal protein in the frontal cortex and hippocampus of AD patients (Jørgensen et al., 1992; Adlard et al., 2005; Jessen et al., 2015; von Holstein-Rathlou et al., 2018; Li et al., 2019). Collectively, all these mechanisms prevent neural damages, and potentially improve the acquisition and retrieval of memory.

#### Neurogenesis

The higher cardiorespiratory fitness and physical activity in aged subjects, have been found to be associated with greater brain structures integrity, and better memory performance (Burzynska et al., 2015). Previous studies confirmed that, exercise training increases gray matter integrity in brain, and volume of brain regions, particularly entorhinal cortex and hippocampus in both human and animal studies (Voss et al., 2013; Ten Brinke et al., 2015; Chieffi et al., 2017; Firth et al., 2018; Tsai S.-F. et al., 2018; Clark et al., 2021), and also, brain white matter of memory-related regions in people with mild cognitive impairment (Amjad et al., 2019). In addition, reduced hippocampal atrophy together with improved memory were reported following 6–12 weeks of exercise training in early stage of Alzheimer’s disease with this finding (Chirles et al., 2017; Ma et al., 2017; Morris et al., 2017). Interestingly, it seems beneficial effects of exercise on brain structures mostly are found in the regions sensitive to neurodegeneration such as the hippocampus and the neocortex in healthy elderly and also adults with Alzheimer’s disease or mild cognitive impairment (Haeger et al., 2019).

How exercise leads in neurogenesis and memory improvement has not been clarified yet. Some researchers believe that intensive exercise training, produces lactate (Scandella and Knobloch, 2019; Nicola and Okun, 2021), and then, lactate stimulates neurons and glia cells proliferation (Steiner et al., 2004; Hirase and Shinohara, 2014; El Hayek et al., 2019), particularly in the hippocampus (Ohia-Nwoko et al., 2014; Elahi et al., 2016). Moreover, lactate induces brain derived neurotropic factor (BDNF) expression in the hippocampus, and then BDNF stimulates neurogenesis (El Hayek et al., 2019).

#### Mitochondrial Biogenesis

An important aspect of exercise training, is the contraction of skeletal muscles, which is associated with enhanced mitochondrial function. There is a bidirectional relationship between the brain and skeletal muscles in a way that both organs benefit from exercise, in order to get adaptation, and in this scenario, mitochondria play pivotal roles in cells survival, metabolism, and oxidative stress (Hood et al., 2019; Burtscher et al., 2021). Studies showed that the number of mitochondria are decreased with aging in healthy adults (Flockhart et al., 2021), and are increased by exercise training (Damirchi et al., 2012; Hood et al., 2019; Huertas et al., 2019; Granata et al., 2021). Exercise training increases antioxidant capacities and the affinity of mitochondria for oxygen (Sun et al., 2016; Hood et al., 2019), also increases proteins involved in energy production and ATP (Bishop et al., 2019; Hood et al., 2019), and finally stimulates mitophagy in skeletal muscles (Bishop et al., 2019; Burtscher et al., 2021), and brain (Navarro et al., 2004; Steiner et al., 2011; Li et al., 2019). Mitochondrial biogenesis after exercise training, is mediated by 5′ adenosine monophosphate-activated protein kinase (AMPK) and peroxisome-proliferator-activated receptor γ coactivator-1α (Short et al., 2005).

On the other hand, mitochondria produce reactive oxygen species (ROS) (Clark and Simon, 2009), in response to intensive acute exercise (Holloway, 2017), which implicates the pathogenesis of several brain disorders (Pesta and Roden, 2017; Zorova et al., 2018). It should be noticed that, exercise-induced ROS levels, can affect redox regulation in brain (Aguiar et al., 2008), and endogenous antioxidant capacities (Quan et al., 2020). In other words, mitochondria are double-edged swords with both beneficial and detrimental effects on memory performance. They not only produce antioxidants molecules, but also increase Ca^++^ and ROS in cytoplasm. Recently, we showed that blocking mitochondrial calcium uniporter, inhibits Ca^++^ neurotoxicity and alleviates cognitive decline in AD model of rats (Nikseresht et al., 2021). There is not clear yet, how it could be possible to keep a balance between antioxidants and ROS in mitochondria (mithohormesis) by exercise training. According to the theory of “Hormesis,” response to exercise might be biphasic, depending on the baseline physical fitness status of individuals (Seifi-Skishahr et al., 2016), and the amounts of ROS (Quan et al., 2020). In other word, exercise might be protective at moderate levels, but, detrimental at high levels in healthy adults (Flockhart et al., 2021). Therefore, regular exercise may prevent the aging-related decline of mitochondrial function.

Taken all together, a lifestyle with moderate regular exercise training, appears to be more useful to improve health by making the balance between oxide and redox state in cells.

#### Neurotrophic Factors

As we mentioned earlier, the relationship between contracted muscles and the brain has been questionable for years. It has been assumed that the number of dendritic connections and neural plasticity is related to the neuroendocrine and humoral alterations promoted by exercise (Isaacs et al., 1992; Santos, 1994). Currently, it has been proposed that, when skeletal muscles are contracted, they start to secrete various proteins, known as myokines into the circulation (Pedersen and Febbraio, 2012). Then, these molecules might elevate neurotrophic factors such as irisin, brain-derived neurotrophic factor (BDNF), and insulin-like growth factor (IGF-1) (Delezie and Handschin, 2018), which all are involved in hippocampal plasticity and long term memory (Lynch et al., 2008; Tanaka et al., 2008; Duzel et al., 2016) following exercise in both animals (Gobbo and O’Mara, 2005; Babaei et al., 2017) and human subjects (Belviranli et al., 2016; Damirchi et al., 2018).

In the next section, we discuss the mechanisms by which, BDNF and irisin might impact on memory and learning.

##### Brain Derived Neurotropic Factor

Exercise training has been known to increase serum BDNF levels, parallel with memory improvement in healthy individuals (Babaei et al., 2013, 2014; Damirchi et al., 2014; Szuhany et al., 2015; Belviranli et al., 2016; Marinus et al., 2019) and roddents (Erickson et al., 2010; Babaei et al., 2017; El Hayek et al., 2019). These findings support the pivotal role for exercise—induced BDNF in brain (Numakawa et al., 2018; Di Liegro et al., 2019). However, there are differences in the baseline level of BDNF considering the subjects’ characteristics. For example, Babaei et al. (2014) showed that long-term habitual exercise in elite athletes is associated with a lower resting level of serum BDNF and better memory. In contrast to elite athletes, a higher level of serum BDNF was detected in subjects diagnosed with metabolic syndrome (MetS) (Damirchi et al., 2014), which might reflect the compensatory role for BDNF.

Collectively, recent studies confirm that, exercise training increases circulatory BDNF levels, regardless of their type, intensity, duration, and subject’s health status (Feter et al., 2019; Marinus et al., 2019). Babaei et al. (2014) showed an increase in serum BDNF level after an acute aerobic/anaerobic exercise in both elite athletes and sedentary subjects. Although Feter et al., 2019 reported that interventions lasting at least 12 weeks with a session duration of 40 min would be the most prominent strategy for increasing BDNF levels in healthy or unhealthy adults (Feter et al., 2019). In contrast, overtraining seems to reduce BDNF level, but upregulates its receptors of “*p75*” and tropomyosin receptor kinase B (TRKB), in intact mice (Xu et al., 2020). Therefore, BDNF releasing system keeps enough sensitivity to be elevated, whenever encountering the different forms of exercise training, although acute exercising is more prominent.

The cellular and molecular mechanisms of exercise-induced BDNF have not been understood yet. El Hayek et al. (2019) suggested that lactate released during exercise by skeletal muscles, crosses the BBB and induces BDNF expression, and activates TRKB signaling in the hippocampus. The function of lactate is dependent on the activation of the transcriptional coactivator; PGC-1 α, and the secreted molecule fibronectin type III domain-containing protein 5 (FNDC5), which both are involved in the upregulation of BDNF expression. Wrann et al. (2013) in an interesting study in mice demonstrated that, aerobic exercise causes production of PGC-1α in muscles, and then PGC-1α induces FNDC5. Then irisin is released by the cleavage of FNDC5 (Lee et al., 2012; Miyamoto-Mikami et al., 2015; Lourenco et al., 2019) and induces BDNF expression through inhibition of histone deacetylase-1, both in human and animal studies (Erickson et al., 2009; Koppel and Timmusk, 2013).

Finally, sustained BDNF levels during exercise, have important roles in cognition through stimulating long-term potentiation, protein phosphorylation, synaptic regeneration (Ji et al., 2005; Tapia-Arancibia et al., 2008), and finally memory improvement in healthy and AD model of rats (Wu et al., 2008; Griffin et al., 2009; Babaei, 2021).

Besides neurotrophic effects which have been discussed above, BDNF also exerts metabotropic roles, and indirectly could influence memory and learning ability, via alleviating systemic insulin resistance in men with MetS (Damirchi et al., 2014), and mitochondrial biogenesis in cultured murine hippocampal neurons (Cheng et al., 2012).

Given the importance of BDNF levels on neuroplasticity and memory, these results support that exercise should be considered as part of rehabilitation programs in different neurodegenerative disorders.

##### Insulin-Like Growth Factor-1

Insulin-like growth factor (IGF-I), is a peptide which is secreted by liver and some other tissues, and stimulates bone growth, and decreases blood glucose levels (Utiger, 2011). Alteration in IGF-I levels, in response to exercise training has inconsistent results in old adults, with or without mild cognitive impairment (Baker et al., 2010; Anderson-Hanley et al., 2017; Tsai C.-L. et al., 2018; Liu et al., 2020; Arazi et al., 2021). Inconsistent results, probably related to various protocols of biochemical assessments, or subjects characteristics. For example, no significant change was found after neither strength nor aerobic exercise in demented patients (Liu et al., 2020). Also a negative correlation was found between endurance exercise and cognitive prognosis when serum IGF-I levels were above 74 ng/ml by Vardy et al. (2007) and Anderson-Hanley et al. (2017). These authors concluded that the higher IGF-I levels, might indicate disease progression, potentially as a compensatory response similar to the higher BDNF levels in metabolic syndrome (Damirchi et al., 2014). Therefore, participants with higher IGF-I levels, may be less likely to benefit from the exercise intervention in either healthy adults or adults with Alzheimer’s disease (Vardy et al., 2007; Anderson-Hanley et al., 2017).

Also increase in both central and peripheral IGF-1 levels following exercise training in rodents (Carro et al., 2000; Trejo et al., 2001; Nakajima et al., 2010; Kim et al., 2019) have been reported. Finally, IGF-1 increases the expression of BDNF (Carro et al., 2000), synaptic plasticity markers such as synaptophysin and postsynaptic density protein-95 in the hippocampus, and represents positive effects on the spatial and aversive memories in healthy rats (Segabinazi et al., 2020).

#### Neurotransmitters (Adrenaline)

Circulatory catecholamines levels have been shown to be increased during exercise training (Kraemer et al., 1999; Sutoo and Akiyama, 2003), and their levels are related to better intermediate and long-term retentions of memory (Winter et al., 2007). Adrenaline is released from the adrenal gland in response to exercise (Kjær, 1998), and has been studied more than other bioamines. Since adrenaline does not cross the BBB (Bradbury, 1993); therefore, it might indirectly affect the brain via activating the vagal nerve (McGaugh et al., 1996), then stimulates noradrenergic inputs of the amygdala (Williams et al., 2000; Miyashita and Williams, 2006), hippocampus (Miyashita and Williams, 2004), and locus coeruleus (Miyashita and Williams, 2006); the main source of brain noradrenaline (McMorris, 2016). Noradrenaline released by locus coeruleus, modulates memory and learning following exercise training both in human (Atzori et al., 2016; Chandler, 2016; Feinstein et al., 2016), and animal studies (Mello-Carpes and Izquierdo, 2013; da Silva de Vargas et al., 2017). Together, these findings suggest an exercise-induced increase of noradrenaline potentiating role on learning and memory.

#### Endocannabinoid

The endocannabinoid system (ECS) is a system of biological lipids, that essentially modulates the functions of the immune, endocrine, and nervous systems (Lu and Mackie, 2016; Zou and Kumar, 2018). In the brain, ECS is involved in various neurophysiological processes including neurogenesis, synaptic plasticity, as well as memory, and emotions (Bisogno and Di Marzo, 2008; Silvestri and Di Marzo, 2013).

Several studies showed that, both acute physical activity (Brellenthin et al., 2017) and regular aerobic exercise, raise endocannabinoid (EC) levels in healthy adults and animal models (Alkadhi, 2018), and elevated plasma levels of EC are potentially associated with long-term beneficial effects on memory and neural plasticity in healthy or adults with major depressive disorders (Coccaro et al., 2018; Stone et al., 2018; Meyer et al., 2019). Moreover, EC reduce anxiety, neuroinflammation, oxidative stress, and brain amyloid-beta deposition (Charytoniuk et al., 2020).

Not only the intensity of physical activity determines the alteration of EC in healthy adults (Feuerecker et al., 2012), but also the duration and subjects characteristics are important as well (Charytoniuk et al., 2020). Some studies showed that chronic exercises might be associated with the upregulation of EC receptor (CB1R) in the hippocampus of mice (Ferreira-Vieira et al., 2014; Brellenthin and Koltyn, 2016). Interestingly, a recent study revealed that isometric handgrip exercise for 3 min, led to major alterations in the EC and its receptor type 1 (Crombie et al., 2017).

Importantly, EC have been known to express BDNF (Sleiman et al., 2016), and then, BDNF regulates the expression of CB1 receptor as well (Maison et al., 2009). In fact, CB1 receptor signaling in glutamatergic neurons increases the BDNF production, and dendritic spine density in the hippocampus and leads to long-term memory in CB1R deficient mice (Wang and Han, 2020).

Consequently, exercise-increased EC and BNDF levels, synergistically improve memory recall and storage in healthy men (Marin Bosch et al., 2021).

Wu et al. (2020) provide new insights into the BDNF/EC-associated modulation of neurotransmission, in the physiological and pathologic processes. They stated that BDNF inhibits the excitatory postsynaptic current (EPSC), presynaptic calcium influx, and exocytosis/endocytosis via activation of the presynaptic CB1 receptors. They also found that BDNF induces the release of endocannabinoids and also retrogradely activates presynaptic CB1Rs via postsynaptic TrkB receptors (Wu et al., 2020).

In this regard, Ferreira et al. (2018) demonstrated that endogenous BDNF is crucial for the cannabinoid-mediated effects on the subventricular zone and dentate gyrus neurogenesis. On the other hand, cannabinoid receptor signaling is also determinant for BDNF actions upon neurogenesis (Ferreira et al., 2018).

Moreover, BDNF attenuates inhibitory transmission by inducing the postsynaptic release of EC, that retrogradely suppress GABA release in the somatosensory cortex (Yeh et al., 2017; Selvam et al., 2018).

Nevertheless, the mutual relationship between physical activity, the endocannabinoid system, and memory performance remains not well discovered and needs proper fulfillment.

#### Myokines

Contracted skeletal muscles following exercise leads to the secretion of various paracrine factors, which are named myokines. Myokines include irisin, myonectin, angiopoietin-like protein (ANGPTL), β-aminoisobutyric acid, fibroblast growth factor 21 (FGF21) (Ingerslev et al., 2017; Cinkajzlová et al., 2018). Myokines are linked with other physiological systems and might impact on their functionality/(Pedersen, 2013; Whitham and Febbraio, 2016). For instance, irisin is a peptide secreted by skeletal muscles, particularly after intermittent high-intensity exercise, and is correlated with glucose and lipids metabolism in skeletal muscles in healthy adults (Huh et al., 2014). Besides metabolic roles, irisin might coordinate locomotion following exercising in healthy rat model (Zhang et al., 2015). Moreover, irisin elevation has been known to be associated with secretion of BDNF, metabolic alterations in human subjects (Huang L. et al., 2019; Arazi et al., 2021), and also facilitation of memory retrieval in male rats (Ding et al., 2006; Babaei et al., 2019). In contrast, finding no correlation between irisin, BDNF, and memory in metabolic syndrome model of rats, indicates that irisin might not be the only mediator for exercise on learning and memory in pathologic conditions such as metabolic syndrome (Babaei et al., 2017).

Another myokine that is elevated after exercise is myonectin. Myonektin is secreted by skeletal muscles and adipose tissue, and induces uptake and oxidation of glucose and fatty acid in healthy adults (Toloza et al., 2018), in response to exercise (Seldin et al., 2012; Toloza et al., 2018). The extent of muscle mass loss and elevated level of myonectin is associated with the severity of cognitive deficits in the Alzheimer’s disease model of mice (Lin et al., 2019).

Since the direct effects of myokines on memory have not been understood yet, here we consider their indirect effects on memory via insulin sensitivity.

#### Improving Insulin Sensitivity

Metabolic syndrome is a complex condition characterized by insulin resistance, hyperglycemia, dyslipidemia, and obesity (Alonso-Gómez et al., 2019), and stands as a risk factor for cognitive decline and Alzheimer’s disease (Razay et al., 2007; Farooqui et al., 2012; Neergaard et al., 2017; Kong et al., 2018). One of the features of metabolic syndrome, is the excess visceral fats producing inflammatory cytokines which are named adipokines. Adipokines are categorized into two groups of pro and anti-inflammatory. Pro-inflammatory adipokines cause oxidative stress and increase inflammation, which consequently leads to memory impairment (Santilli et al., 2017; Funcke and Scherer, 2019). On the other hand, exercise has been known to reduce pro-inflammatory, but increase anti-inflammatory adipokines and prevents the progression of metabolic syndrome toward type II diabetes (Babaei et al., 2015). In addition, besides alteration in adipokine levels, exercise alleviates cognitive decline in middle-aged men with metabolic syndrome (Damirchi et al., 2014), by epigenetic modulation in cell metabolism (Sjøberg et al., 2017). For instance, the first beneficial effect of exercise or lipolytic action involves the phosphorylation and activation of AMPK in healthy mice (Huang J. et al., 2019; Yoon et al., 2019). AMPK is a fuel-sensing enzyme, which is activated after the increase in the cellular ratio of AMP relative to ATP. Animal studies on healthy mice revealed that AMPK also mediates mitochondrial biogenesis (Jørgensen et al., 2007), angiogenesis (Ouchi et al., 2005), BDNF production, and therefore reverse memory deficits (Kim and Leem, 2016). Moreover, exercise directly activates the autophagy through up-regulating the AMPK-SIRT1 signaling pathway (Huang J. et al., 2019), and remove abnormal proteins responsible for neurodegenerative diseases in mouse (Osellame and Duchen, 2014; Lin et al., 2020). Elevated AMPK exerts insulin sensitivity in healthy rats (Zhang et al., 2011), thus attenuates the progression of neurodegeneration in human and animal models with AD (Watson and Craft, 2004).

The second beneficial effect of exercise, takes place via activating phosphoinositide 3-kinase and translocating the glucose transporter type 4 into the skeletal muscles (Vega et al., 2017), and the third mechanism mediated by adipokines (Beavers et al., 2010). One of the pro-inflammatory adipokines, is TNF-α which leads to insulin resistance, and induces a chronic state of local inflammation in rats with diabetes or metabolic syndrome (Samarghandian et al., 2016; Kouhestani et al., 2018). TNF-α also contributes to cognitive decline in AD patients by elevating oxidative stress and apoptosis (Perry et al., 2007; Janelsins et al., 2008). Both aerobic and exercise training have been demonstrated to be efficient in reducing TNF-α in young adults and suppressing neuroinflammation (Flynn et al., 2003; Forti et al., 2017; Monteiro-Junior et al., 2018).

On the other hand, reduction in anti-inflammatory adipokines, more notably, adiponectin is associated with metabolic syndrome in a rat model (Damirchi et al., 2010), coronary heart disease in adult patients (Lindberg et al., 2017), and cognitive decline in adults with AD (Teixeira et al., 2013). Adiponectin receptor-1 knockdown mice exhibited spatial learning and memory impairment (Kim et al., 2017), however exercise training exerts anti-inflammatory and insulin-sensitizing effects via elevating adiponectin and inhibiting TNFα and IL-6 (Tore et al., 2007; Rizzo et al., 2020). Administration of exogen adiponectin improves learning and memory (Tore et al., 2007), similar to the exercise-induced elevated adiponectin level (Martinez-Huenchullan et al., 2018; Pousti et al., 2018; Diniz et al., 2019; Parastesh et al., 2019) in animal studies.

Considering more than 800 adipokines, it needs more studies to clarify the exact roles of these molecules on memory following exercise training.

#### Epigenetic Mechanisms

Acute and regular exercise training induces both short and long-term epigenetic regulations, creating a “functional genome” that consequently leads to adaptation during the active life span of individuals. For example, memory-boosting effects of exercise training could be partly mediated by DNA methylation (Deibel et al., 2015; Kim and Kaang, 2017), histone acetylation (Barrett and Wood, 2008; Fernandes et al., 2017), as well as up and downregulation of microRNA (Fernandes et al., 2017; Grazioli et al., 2017). Recently, an increase in histone acetylation of the BDNF and the expression of immediate early genes of *c-fos* and *Arc*, parallel with improvement in plasticity and memory consolidation, storage, and retrieval in senescence-accelerated mice following aerobic exercise have been reported (Maejima et al., 2018, 2021).

In contrast with regular exercise training, an acute exercise had no significant epigenetic change in basal levels of plasma BDNF considering histone acetylation in healthy amateur runners (da Silveira et al., 2017).

Taken altogether, epigenetic modification of exercise is incomplete, and needs to be evaluated considering different protocols timing and new candidate genes.

##### MicroRNAs

MicroRNAs (miRNAs) are small, single-strand non-coding RNAs that play pivotal roles in the post-transcriptional regulation of genes responsible for various physiological functions (Baek et al., 2008). A growing body of evidence in human and animal studies has shown that exercise alters blood levels of several miRNAs (Flowers et al., 2015; Gomes et al., 2015; Xu et al., 2015), and these small molecules modulate communication between the brain and muscles. However, the patterns of miRNAs regulation in response to exercise training is very complicated. Some of them are upregulated in response to acute exercise (miR-146a, miR-222), but some are increased in response to sustained training (miRNA-20), and other remain non-responsive (miR-133a, miR-210, and miR-328) (Baggish et al., 2011). One of the broadly studied miRNA in response to exercise is miR-132. This mi RNA is involved in memory formation and synaptic plasticity (Scott et al., 2012; Hansen et al., 2013; Wang et al., 2013). For instance, Radom-Aizik et al. (2012) reported a rapid elevation in circulating levels of miR-132 in healthy men in response to an acute intermittent exercise, but a reduction in trained human subjects (de Gonzalo-Calvo et al., 2015, 2018). Meanwhile, an animal study carried out by Dong et al. (2018) showed increased miR-132 level in the hippocampus of the mouse model of AD after aerobic exercise, parallel with memory improvement. In contradictory, Smith et al. (2015) and Hernandez-Rapp et al. (2016) reported a reduction in the expression of miR-132 in the transgenic mice model of AD. The inconsistency in literature, might be related to the model and stages of AD development, and also the sensitivity of biochemical assessments used for mi RNA.

In conclusion, exercise training could mitigate the aging-induced memory decline by regulating the hippocampal expression of miR-132 in the AD mice model (Dong et al., 2018), miR-21 in mice with traumatic brain injury (Hu et al., 2015), miR-34a and miR-124 in rats with cognitive impairment (Pan-Vazquez et al., 2015; Kou et al., 2017).

Taken all together, it seems the bioinformatic analysis is required to summarize the panel of various miRNA in response to exercise training, rather than a single molecule.

## Conclusion

In conclusion, this review provides an affordable and effective method to improve cognitive function in all ages, particularly the elderly who are most vulnerable to neurodegenerative disorders. In spite of the limitations of this review, it is suggested that frequent moderate aerobic activity is associated with improved neurocognitive performance for elderly people. Improved brain circulation, neurotrophic factors, mitochondrial biogenesis, and the release of numerous signaling molecules, including myokines and adipokines in response to regular exercise might be involved in the neuroprotective mechanisms of exercise training. Currently, among various mechanisms, irisin/BDNF signaling seems to stand at the core of exercise facilitatory effects on learning and memory. These molecular signalings anticipate better understanding of mechanisms that will enable the development of pharmaceuticals, particularly for those who are activity limited (coma, spinal cord injury, etc.).

The strengths of this review is describing updated findings on various exercise modalities and memory performance which uncovers molecular and cellular linkages critical in studies of aging and neurodegeneration. However, the limitations of the present review are focusing only on the selected possible mechanisms including some of the important mediators and signaling pathways.

Future studies need to find standard protocol for exercise intensity, and also adequate follow-ups to consider the maintenance of neurocognitive effects of exercise.

## Author Contributions

PB participated in the design and coordination of the manuscript, drafted, and revised it. HA participated in drafting the manuscript and managing references, providing the graphical abstract. Both authors have read and approved the final version of the manuscript and agreed with the order of presentation of the authors.

## Conflict of Interest

The authors declare that the research was conducted in the absence of any commercial or financial relationships that could be construed as a potential conflict of interest.

## Publisher’s Note

All claims expressed in this article are solely those of the authors and do not necessarily represent those of their affiliated organizations, or those of the publisher, the editors and the reviewers. Any product that may be evaluated in this article, or claim that may be made by its manufacturer, is not guaranteed or endorsed by the publisher.
